# Supplementary material for: Decidualization-empowered ECM hydrogel integrating sustained Tβ4 release drives endometrial regeneration in intrauterine adhesions
Source: Nat Commun. 2026 Jan 21;17:1910. doi: 10.1038/s41467-026-68677-w (PMC12923885; doi:10.1038/s41467-026-68677-w)
Supplement: Supplementary file 1 — Supplementary Information [file 41467_2026_68677_MOESM1_ESM.pdf]

Supplementary Information

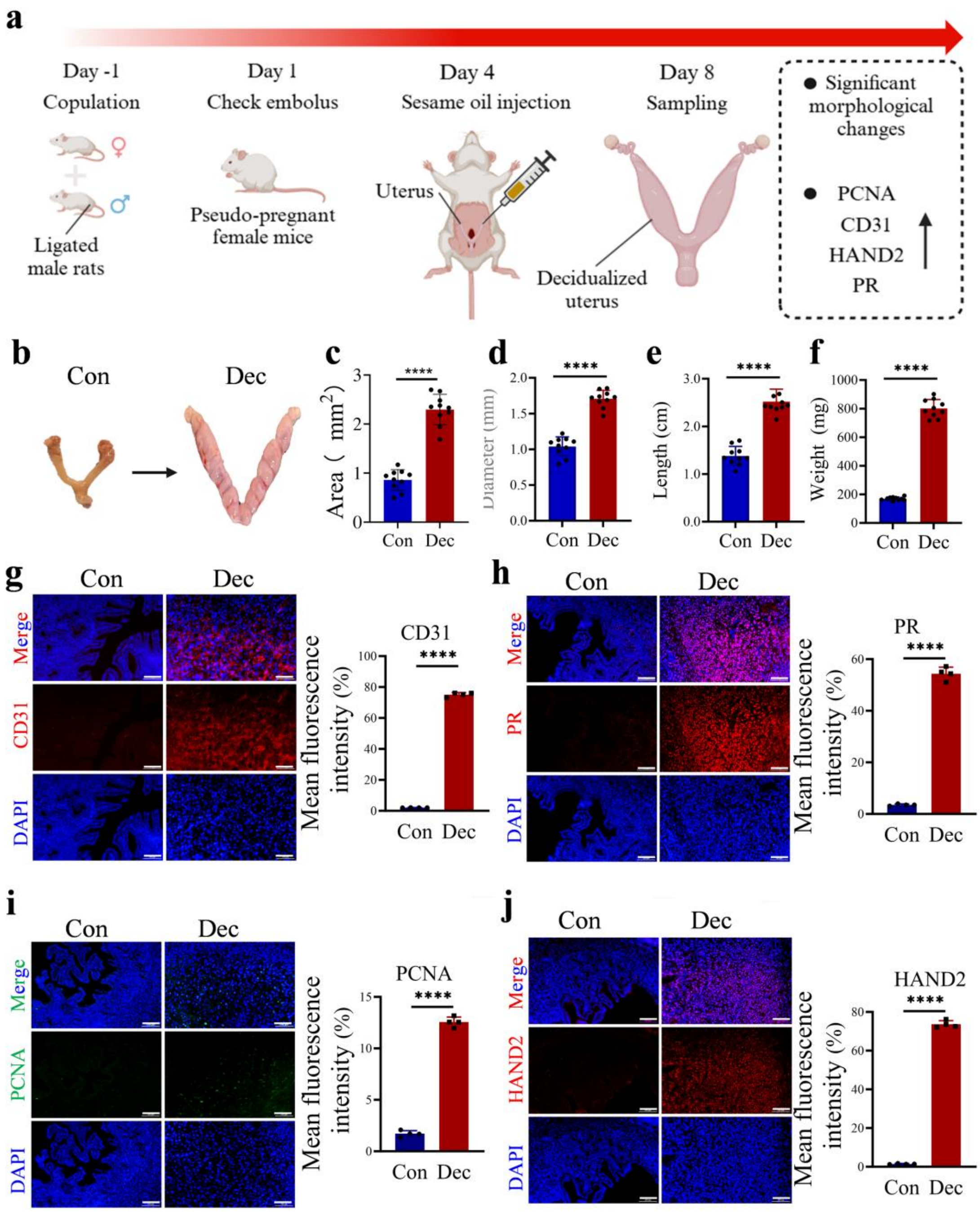

Supplementary Figure 1 | Establishment and characterization of a mouse uterine decidualization model.

a, Schematic of the experimental workflow for inducing uterine decidualization in pseudopregnant mice (Created in BioRender. Zhaowei, Y. (2025) https://BioRender.com/ul4iqzn). **b,** Representative macroscopic images of uteri from control (Con) and decidualized (Dec) mice. **c–f,** Quantification of uterine cross-sectional area (**c**), axial diameter (**d**), length (**e**), and weight (**f**). **g–j,** Immunofluorescence analysis and quantification of key decidualization-associated markers: the endothelial marker CD31 (red) (**g**), Progesterone Receptor (PR; red) (**h**), the proliferation marker Proliferating Cell Nuclear Antigen (PCNA; green) (**i**), and the decidualization transcription factor

HAND2 (red) (**j**). Cell nuclei are counterstained with DAPI (blue). Scale bars, 100 µm. In all quantitative plots (**c–f, g–j**), data are presented as scatter dot plots from n = 6 biologically independent animals, with data shown as mean ± s.d. Statistical significance was determined by an unpaired, two-tailed Student's t-test; $***P < 0.001$. Detailed statistical analyses, including exact P values, test statistics (F values and degrees of freedom), and 95% confidence intervals, are provided in the Source Data file.

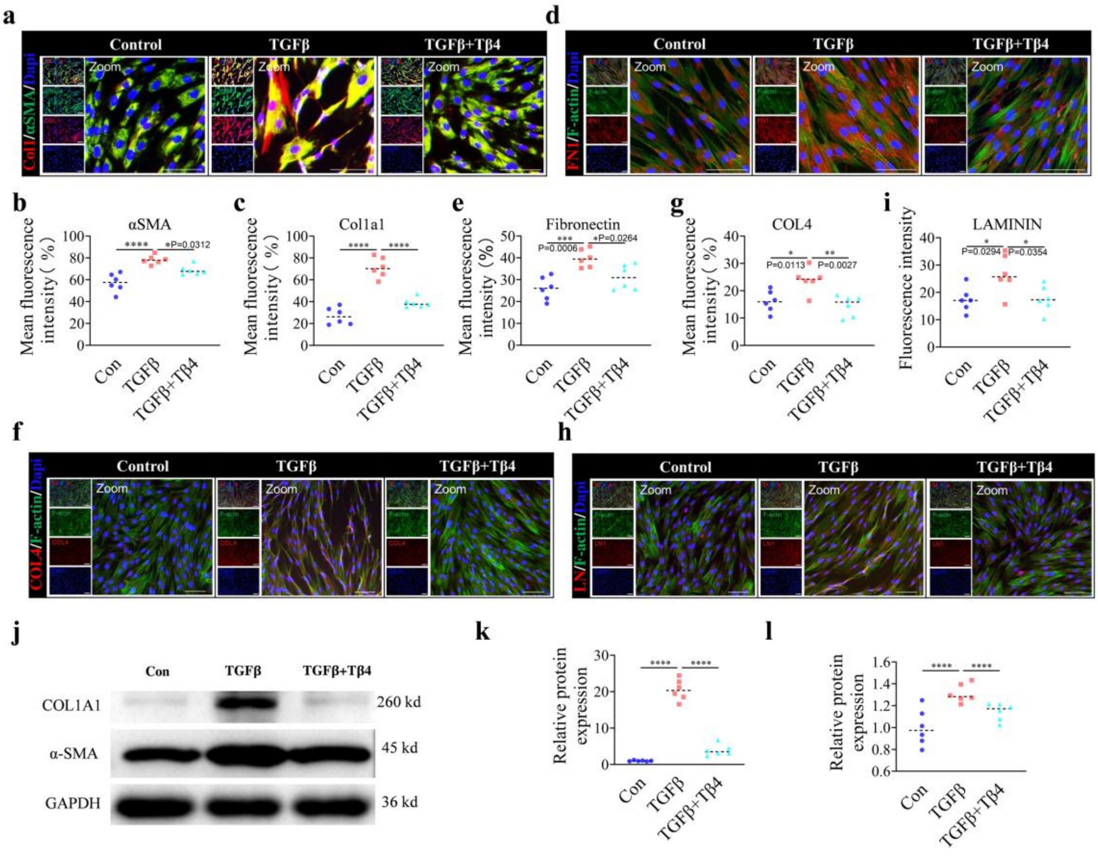

Supplementary Figure 2 | Tβ4 suppresses TGF-β1-induced fibrotic activation in human endometrial stromal cells (hESCs) *in vitro*.

Human endometrial stromal cells were stimulated with TGF-β1 (10 ng/mL) for 48 h to induce a fibrotic phenotype, with or without co-treatment with Tβ4. **a,** Representative immunofluorescence images co-staining for the myofibroblast marker α-Smooth Muscle Actin (αSMA; green) and the key fibrosis marker Collagen Type I Alpha 1 (COL1A1; red). **b, c,** Quantification of the mean fluorescence intensity for αSMA (**b**) and COL1A1 (**c**). **d, f, h,** Representative immunofluorescence images for additional extracellular matrix (ECM) proteins: Fibronectin (FN1; red) (**d**), Collagen Type IV (COL4; red) (**f**), and Laminin (LN; red) (**h**). In these panels, the actin cytoskeleton is visualized with phalloidin (F-actin; green). **e, g, i,** Corresponding quantification of mean fluorescence intensity for FN1 (**e**), COL4 (**g**), and Laminin (**i**). **j,** To provide orthogonal validation, protein levels of key fibrotic markers were assessed by western blot. Representative western blot bands for COL1A1 and α-SMA are shown, with GAPDH serving as a loading control. **k, l,** Corresponding densitometric quantification for COL1A1 (**k**) and α-SMA (**l**). In all immunofluorescence images, cell nuclei are counterstained with DAPI (blue). Scale bars, 100 µm.

In all quantitative plots, data are presented as scatter plots with mean ± s.d. from n = 6 biologically independent experiments. Statistical significance was determined by one-way ANOVA with Tukey's post hoc test; **P* < 0.05, ***P* < 0.01, ****P* < 0.001 vs. the TGF-β1 group. Detailed statistical analyses, including exact P values, test statistics (F values and degrees of freedom), and 95% confidence intervals, are provided in the Source Data file.

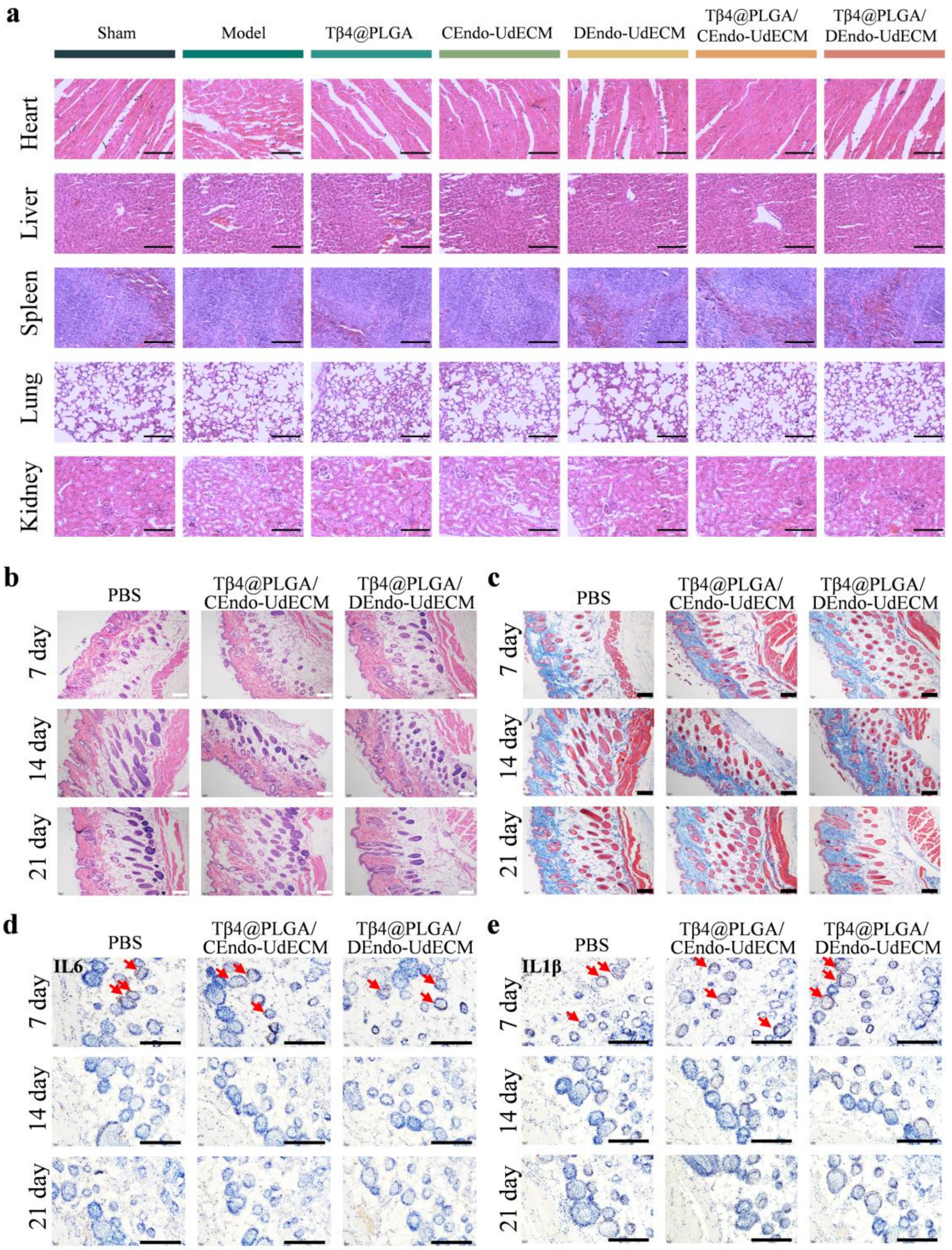

Supplementary Figure 3 | Systemic and local biocompatibility assessment of the hydrogel formulations.

a, Representative haematoxylin and eosin (H&E) staining of major organs (heart, liver, spleen, lung, kidney) 14 days post-treatment reveals no histopathological abnormalities or signs of systemic toxicity in any group. **b, c,** Local tissue response following subcutaneous implantation over 21 days. H&E staining (**b**) shows a minimal and resolving inflammatory infiltrate, while Masson's trichrome

staining (**c**) indicates only thin fibrous capsule formation, demonstrating excellent tissue integration. **d, e,** Immunohistochemistry for pro-inflammatory cytokines IL-6 (**d**) and IL-1β (**e**) at the implantation site. Expression levels were transient and remained comparable to the PBS control, confirming the absence of a material-specific chronic inflammatory response. Scale bars, 100 µm.

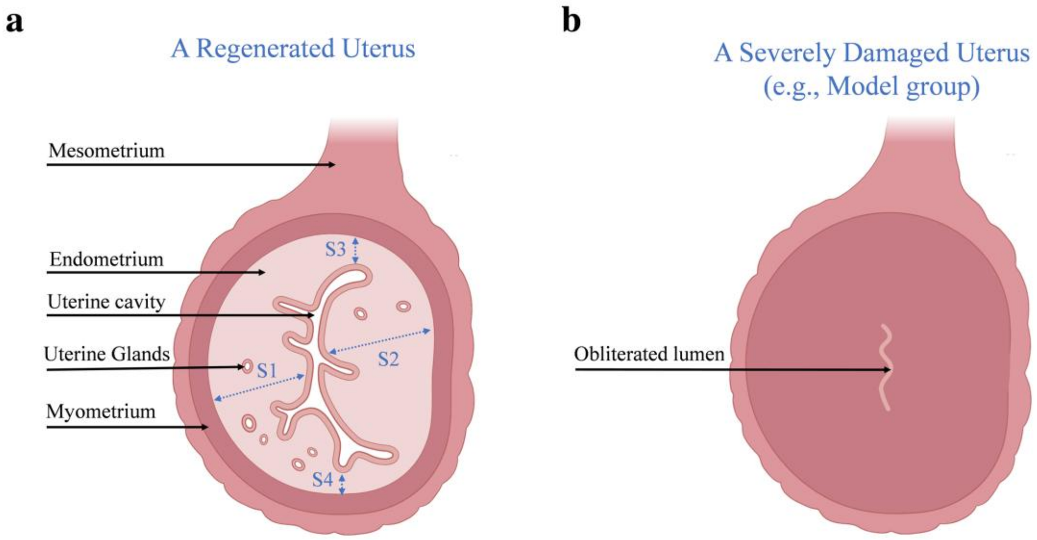

Supplementary Figure 4 | Methodology for the quantification of endometrial thickness. (Created in BioRender. Zhaowei, Y. (2025) https://BioRender.com/ul4iqzn).

a, Schematic of a regenerated uterus with a patent (open) lumen. Endometrial thickness was defined as the average of four equidistant measurements (S1–S4), each representing the perpendicular distance from the myometrial-endometrial junction to the luminal surface. **b,** Schematic of a severely damaged uterus, characteristic of the Model group, where the uterine cavity is obliterated by fibrotic tissue and a distinct endometrial layer is absent. For these samples, a meaningful thickness measurement was biologically impossible; therefore, a value of zero was assigned to quantitatively represent a complete failure of structural regeneration.

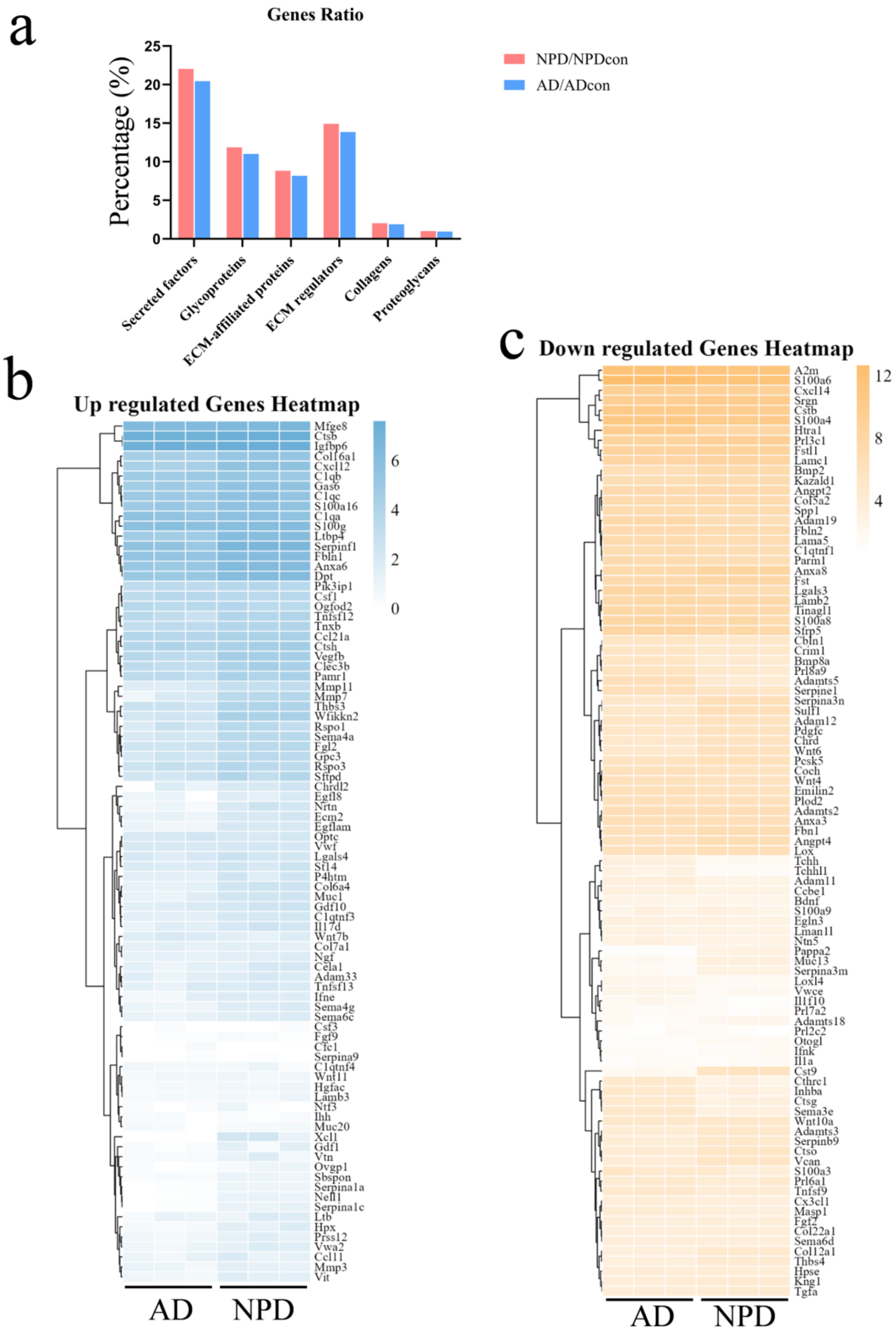

Supplementary Figure 5 | Comparative proteomic analysis validates the artificially induced decidualization model against the natural pregnancy benchmark.

a, Bar chart comparing the distribution of differentially expressed matrisome-affiliated proteins across major functional categories. The analysis shows a high degree of similarity in the proportional representation of these categories between the artificially decidualized versus control (AD/ADcon) and the natural pregnancy decidualization versus control (NPD/NPDcon) datasets. **b,**

Heatmap with hierarchical clustering of significantly upregulated matrisome-affiliated proteins, comparing their expression levels in AD and NPD samples. **c,** Heatmap with hierarchical clustering of significantly downregulated matrisome-affiliated proteins in AD and NPD samples. In heatmaps (**b, c**), color intensity represents the relative protein expression level (Z-score).

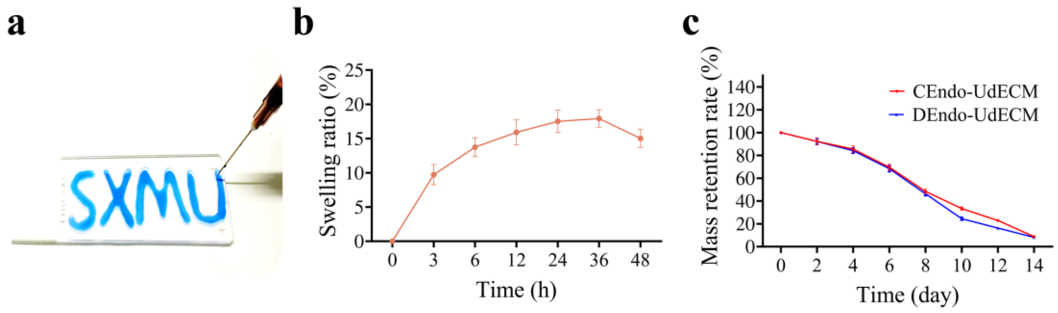

Supplementary Figure 6 | Physicochemical characterization of the UdECM hydrogels.

a, Demonstration of the injectability of the DEndo-UdECM hydrogel precursor solution. The solution was stained with methylene blue for visualization and extruded through a 25-gauge needle to form stable, well-defined letters. **b,** swelling kinetics of the crosslinked DEndo-UdECM hydrogel in phosphate-buffered saline (PBS) at 37 ° Cover 48 h. **c,** Comparative *in vitro* degradation profiles of hydrogels derived from non-decidualized (CEndo-UdECM) and decidualized (DEndo-UdECM) uterine ECM over 14 days. Data in **b**, **c** are presented as mean ± s.d. (n = 3 biologically independent samples). Source data are provided as a Source Data file.

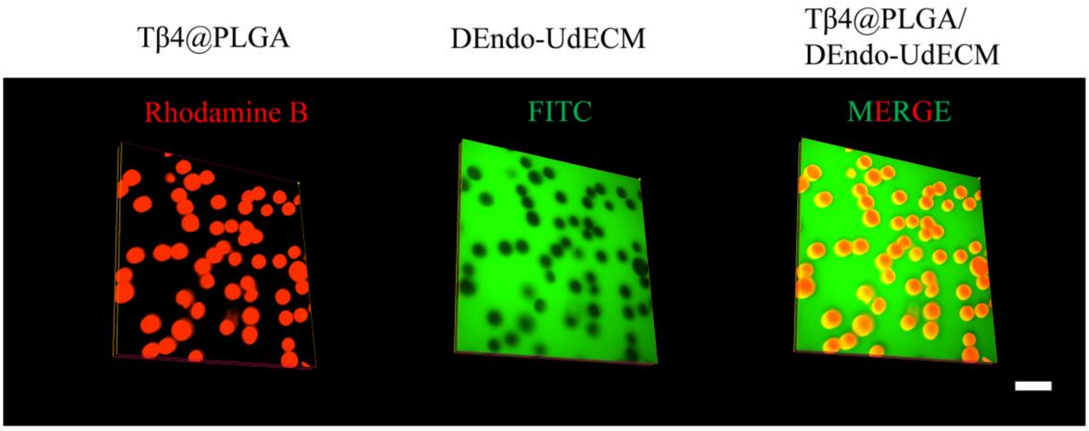

Supplementary Figure 7 | Three-dimensional confocal analysis of microsphere distribution within the composite hydrogel.

Representative 3D renderings from confocal z-stack imaging of the DEndo-UdECM/Tβ4@PLGA composite hydrogel. Tβ4@PLGA microspheres were labeled with Rhodamine B (red) and the DEndo-UdECM hydrogel matrix was labeled with FITC (green). Individual channels for

microspheres and hydrogel, along with the merged image, are shown. The volumetric reconstruction confirms the homogeneous dispersion of the microspheres throughout the hydrogel matrix, definitively resolving ambiguities that can arise from 2D projection imaging. Scale bar,100μm.

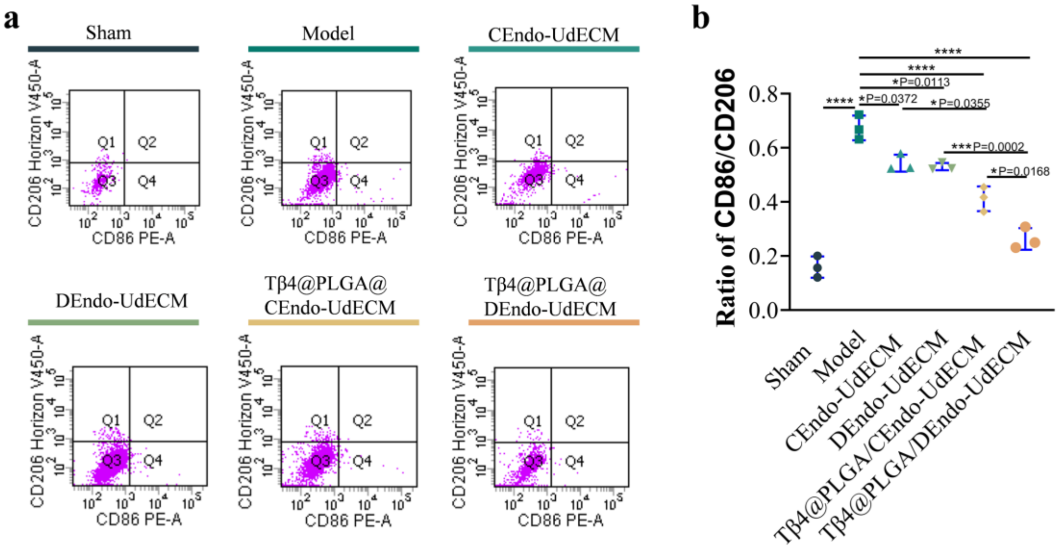

Supplementary Figure 8 | Flow cytometric quantification confirms the DEndo-UdECM/Tβ4@PLGA hydrogel skews macrophage polarization towards a pro-regenerative M2 phenotype.

a, Representative flow cytometry dot plots of single-cell suspensions from endometrial tissues of different treatment groups, 14 days post-surgery. Cells were pre-gated on the F4/80^{+} macrophage population and analyzed for the expression of the M1 marker CD86 (PE-A channel) and the M2 marker CD206 (Horizon V450-A channel). Quadrants delineate M1-like (Q2: CD86^{+}CD206^{-}), M2-like (Q4: CD86^{-}CD206^{+}), and other populations. **b,** Quantification of the M1/M2 ratio, calculated from the percentage of CD86^{+} versus CD206^{+} cells within the F4/80^{+} gate for each group. The significant decrease in this ratio in the Tβ4@PLGA@DEndo-UdECM group indicates a strong shift towards M2 polarization. Data are presented as mean ± s.d. (n = 3 biologically independent animals). *$P < 0.05$, **$P < 0.01$, ***$P < 0.001$. Detailed statistical analyses, including exact P values, test statistics (F values and degrees of freedom), and 95% confidence intervals, are provided in the Source Data file.

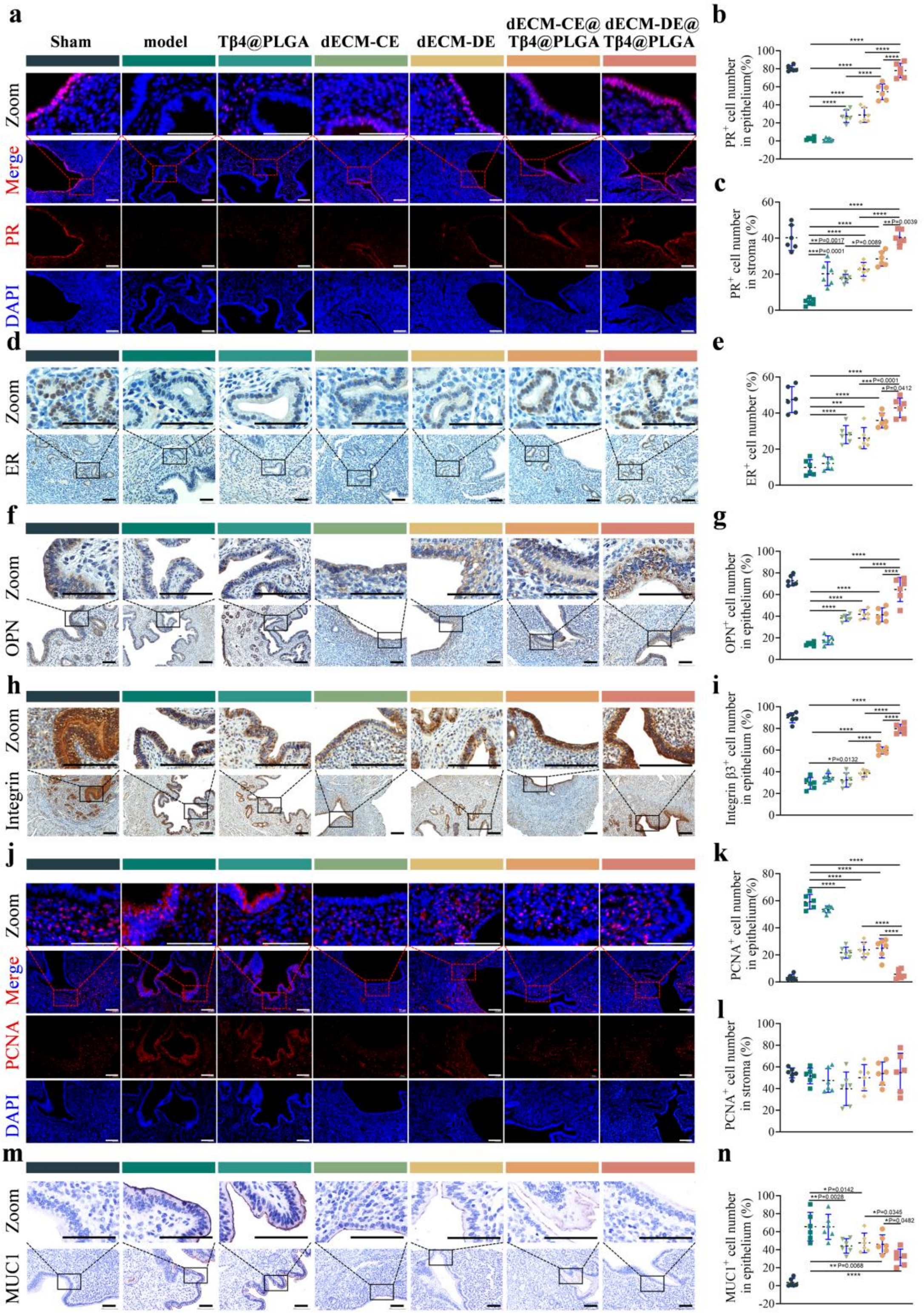

Supplementary Figure 9 | The DEndo-UdECM/Tβ4@PLGA hydrogel restores a receptive endometrial phenotype.

a–n, Analysis of key endometrial receptivity and proliferation markers in uterine tissue 14 days post-treatment. **a–c,** Immunofluorescence analysis of Progesterone Receptor (PR; red) expression (**a**) and corresponding quantification of PR-positive cells in the luminal epithelium (**b**) and stroma (**c**). **d, e,** Immunohistochemical analysis of Estrogen Receptor (ER) expression (**d**) and

quantification in the stroma (**e**). **f, g,** Immunohistochemical analysis of Osteopontin (OPN) expression (**f**) and quantification in the luminal epithelium (**g**). **h, i,** Immunohistochemical analysis of Integrin β3 expression (**h**) and quantification in the luminal epithelium (**i**). **j–l,** Immunofluorescence analysis of Proliferating Cell Nuclear Antigen (PCNA; red) expression (**j**) and quantification in the luminal epithelium (**k**) and stroma (**l**). **m, n,** Immunohistochemical analysis of Mucin 1 (MUC1) expression (**m**) and quantification in the luminal epithelium (**n**). In immunofluorescence images (**a, j**), cell nuclei are counterstained with DAPI (blue). For all representative images, high-magnification insets (Zoom) of the boxed areas are provided. Scale bars, 100 μm. In all quantitative plots (**b, c, e, g, i, k, l, n**), data are presented as scatter dot plots from n = 6 biologically independent animals, with data shown as mean ± s.d. Statistical significance was determined by one-way ANOVA with Tukey's post hoc test; $^{*}P < 0.05$, $^{**}P < 0.01$, $^{***}P < 0.001$. All comparisons are against the Model group unless otherwise indicated by brackets. Detailed statistical analyses, including exact P values, test statistics (F values and degrees of freedom), and 95% confidence intervals, are provided in the Source Data file.

Supplementary Table 1 | antibody

Primary/secondary antibodies	Dilution Factor	Manufacturer	Cat.No
Mouse polyclonal to PCNA antibody	1:200	Proteintech	60097-1-lg
Rabbit monoclonal to Integrin β3 antibody	1:200	Abmart	T55237
Rabbit polyclonal to MUC1 antibody	1:200	Abcam	ab109185
Rabbit monoclonal to OPN antibody	1:200	Abmart	T55333
Rabbit polyclonal to ER antibody	1:200	Abcam	ab32063
Rabbit polyclonal to PR antibody	1:200	Abcam	ab16661
Rabbit polyclonal to HAND2 antibody	1:200	Abcam	ab200040
Mouse polyclonal to Collagen Ⅰ antibody	1:200	Cell Signaling	66948
Rabbit polyclonal to Collagen Ⅳ antibody	1:200	Abcam	ab6586
Rabbit polyclonal to α-SMA antibody	1:200	Cell Signaling	19245
Rabbit polyclonal to Fibronectin antibody	1:200	Abcam	ab32419
Rabbit polyclonal to FoxA2 antibody	1:200	Cell Signaling	8186
Mouse polyclonal to pan CK antibody	1:200	Abcam	ab7753
Mouse polyclonal to CD34 antibody	1:200	Abcam	ab6330
Rabbit polyclonal to ALDH1A1 antibody	1:200	Abcam	ab52492
Rabbit polyclonal to CD86 antibody	1:200	Cell Signaling	19589
Rabbit polyclonal to Liver Arginase antibody	1:200	Abcam	ab315110
Rabbit polyclonal to Mannose Receptor (CD206) antibody	1:200	Abcam	ab300621
Rabbit polyclonal to iNOS antibody	1:201	Abcam	ab283655
Rabbit polyclonal to Ki67 antibody	1:200	Abcam	ab16667
Mouse polyclonal to TGFβ1 antibody	1:200	Santa	sc130348
Rabbit polyclonal to GSDMD antibody	1:1000	Abmart	TA4012F
Rabbit polyclonal to P-smad3 antibody	1:1000	Affinity	AF3362
Rabbit polyclonal to IL-6 antibody	1:200	Abcam	ab290735
Rabbit polyclonal to IL-1 β antibody	1:1000	Cohesion	CQA6579
Rabbit polyclonal to GSDMD-NT antibody	1:1000	Cohesion	CQA3563
Rabbit polyclonal to IL-18 antibody	1:1000	Cohesion	CQA2028
Rabbit polyclonal to Caspase1-P10-P12 antibody	1:1000	Abcam	ab179515
Rabbit polyclonal to GAPDH antibody	1:10000	Bioss	bs-10900R
Dylight 594 Conjugated AffiniPure Donkey Anti-Rabbit IgG (H+L)	1:200	Boster Bio	BA1147

DyLight 488 Conjugated AffiniPure Donkey Anti-Rabbit IgG (H+L)	1:200	Boster Bio	BA1146
Dylight 594 Conjugated AffiniPure Donkey Anti-Mouse IgG (H+L)	1:200	Boster Bio	BA1148
DyLight 488 Conjugated AffiniPure Donkey Anti-Mouse IgG (H+L)	1:200	Boster Bio	BA1145
Goat Anti-Rabbit IgG H&L/HRP	1:10000	Bioss	Bs-0295G-HRP
APC/Cy7-conjugated anti-mouse CD45	1:200	BioLegend	103115
APC-conjugated anti-mouse F4/80	1:200	BioLegend	123115
PE-conjugated anti-mouse CD86	1:100	BioLegend	159204
Brilliant Violet 421ö -conjugated anti-mouse CD206 (MMR)	1:100	BioLegend	141717

Supplementary Table 2 | Postnatal Survival Rate (%)

Postnatal Survival Rate (%)						
Sham	Model	Tβ 4@PLGA	CEndo-UdECM	DEndo-UdECM	CEndo-UdECM/Tβ 4@PLGA	DEndo-UdECM/T β4@PLGA
100	100	100	100	100	100	100
