# Supplementary material for: Decidualization-empowered ECM hydrogel integrating sustained Tβ4 release drives endometrial regeneration in intrauterine adhesions
Source: Nat Commun. 2026 Jan 21;17:1910. doi: 10.1038/s41467-026-68677-w (PMC12923885; doi:10.1038/s41467-026-68677-w)
Supplement: Supplementary file 2 — Description of Additional Supplementary Files [file 41467_2026_68677_MOESM2_ESM.pdf]

Title: Supplementary Movie 1 | Rotational 3D view of the composite hydrogel.

Description: Movie corresponding to Supplementary Supplementary Figure 7. A three-dimensional rotational rendering generated from confocal z-stack data, visualizing the homogeneous distribution of Rhodamine B-labeled Tβ4@PLGA microspheres (red) within the FITC-labeled DEndo-UdECM hydrogel matrix (green). The rotation provides a comprehensive view of the material's internal architecture from all angles, confirming the absence of significant microsphere aggregation.
