# Supplementary material for: Decidualization-empowered ECM hydrogel integrating sustained Tβ4 release drives endometrial regeneration in intrauterine adhesions
Source: Nat Commun. 2026 Jan 21;17:1910. doi: 10.1038/s41467-026-68677-w (PMC12923885; doi:10.1038/s41467-026-68677-w)
Supplement: Supplementary file 4 — Reporting summary [file 41467_2026_68677_MOESM4_ESM.pdf]

nature portfolio

Corresponding author(s): Jun Xie, Yuxiang Liang, Liping Li, Qizhi Shuai and zhizhen liu

Last updated by author(s): May 13, 2025

Reporting Summary

Nature Portfolio wishes to improve the reproducibility of the work that we publish. This form provides structure for consistency and transparency in reporting. For further information on Nature Portfolio policies, see our Editorial Policies and the Editorial Policy Checklist.

Statistics

For all statistical analyses, confirm that the following items are present in the figure legend, table legend, main text, or Methods section.

n/a	Confirmed	
☐	☒	The exact sample size (*n*) for each experimental group/condition, given as a discrete number and unit of measurement
☐	☒	A statement on whether measurements were taken from distinct samples or whether the same sample was measured repeatedly
☐	☒	The statistical test(s) used AND whether they are one- or two-sided<br>*Only common tests should be described solely by name; describe more complex techniques in the Methods section.*
☐	☒	A description of all covariates tested
☐	☒	A description of any assumptions or corrections, such as tests of normality and adjustment for multiple comparisons
☐	☒	A full description of the statistical parameters including central tendency (e.g. means) or other basic estimates (e.g. regression coefficient) AND variation (e.g. standard deviation) or associated estimates of uncertainty (e.g. confidence intervals)
☐	☒	For null hypothesis testing, the test statistic (e.g. *F*, *t*, *r*) with confidence intervals, effect sizes, degrees of freedom and *P* value noted<br>*Give P values as exact values whenever suitable.*
☒	☐	For Bayesian analysis, information on the choice of priors and Markov chain Monte Carlo settings
☒	☐	For hierarchical and complex designs, identification of the appropriate level for tests and full reporting of outcomes
☒	☐	Estimates of effect sizes (e.g. Cohen's *d*, Pearson's *r*), indicating how they were calculated

Our web collection on statistics for biologists contains articles on many of the points above.

Software and code

Policy information about availability of computer code

Data collection

Spectrophotometer (Nanodrop 2000, Thermo Fisher Scientific, USA) was used for DNA concentration quantification.
Inverted fluorescence microscope (Nikon Ti2, Japan) was used for image capturing in immunofluorescence staining (ECM components, α-SMA, COL1A1, Ki67, FOXA2, CD34, Pan-CK, ALDH1A1, TGF-β, CD86, Liver arginase, PR, ERα, integrin β3, OPN, PCNA, MUC1), EdU incorporation assays, and Live/Dead staining assays.
UltiMate 3000 RSLCnano liquid chromatography system (Thermo Fisher Scientific) coupled to a timsTOF Pro2 trapped ion mobility mass spectrometer (Bruker Daltonik) was used for LC-MS/MS analysis of peptides.
Scanning electron microscope (SEM, ZEISS GeminiSEM 300, Germany) was used for observing the surface morphology and size of Tβ4@PLGA microspheres and the internal porous morphology of hydrogels.
Dynamic light scattering instrument (DLS, Malvern Mastersizer 2000, UK) was used for measuring the particle size distribution of Tβ4@PLGA microspheres.
Tβ4 ELISA Kit (JL19928, JONLNBIO, China) along with a microplate reader (model not specified, but implied for ELISA) was used for determining Tβ4 concentration in drug loading and in vitro release studies.
Rotational rheometer (Anton Paar MCR 302, Austria) was used for rheological measurements (storage modulus G′ and loss modulus G′′).
Inverted microscope (Nikon Ti2, Japan) was used for image capturing in scratch assays and hUVEC tube formation assays. (Note: Though specified as "inverted microscope", likely the same Nikon Ti2 fluorescence microscope was used for brightfield in these assays).
Light microscope (specific model not detailed, but implied for IHC where DAB chromogen is used) was used for image capturing in immunohistochemistry (PR, ERα, integrin β3, OPN, PCNA, MUC1).
Chemiluminescence imaging system (Tanon 4600, Shanghai) was used for Western blot band visualization and capturing.
High-resolution spinning-disk confocal microscope (Evident SIM-ultimate) was used for capturing Z-stack images of the composite hydrogel.
BD FACSAria™ Fusion Flow Cytometers (BD Biosciences, USA) was used for flow cytometric.

Data analysis	• Raw DIA data files from LC-MS/MS were processed using Spectronaut™ Pulsar X software (Biognosys AG) for peptide identification and label-free quantification.<br>• Differential protein expression analysis, Gene Ontology (GO) functional enrichment analysis, and Kyoto Encyclopedia of Genes and Genomes (KEGG) pathway analysis for proteomic data were performed using R statistical software and online tools such as DAVID or Metascape.<br>• Quantification of mean fluorescence intensity, wound closure rate/migration distance, EdU proliferation rate, percentage of Ki67-positive cells, number of nodes in tube formation assays, endometrial thickness, percentage of fibrotic area, quantitative analysis of IHC/IF staining (e.g., mean optical density or percentage of positive area), and Western blot band intensities were performed using ImageJ software (NIH).<br>• Statistical analyses were performed using GraphPad Prism 8.0 software (GraphPad Software, La Jolla, CA, USA).

For manuscripts utilizing custom algorithms or software that are central to the research but not yet described in published literature, software must be made available to editors and reviewers. We strongly encourage code deposition in a community repository (e.g. GitHub). See the Nature Portfolio guidelines for submitting code & software for further information.

Data

Policy information about availability of data

All manuscripts must include a data availability statement. This statement should provide the following information, where applicable:

- Accession codes, unique identifiers, or web links for publicly available datasets
- A description of any restrictions on data availability
- For clinical datasets or third party data, please ensure that the statement adheres to our policy

The data and statistical evaluations supporting the findings of this study are available within the article, the Supplementary Information, and the Source Data file. The mass spectrometry proteomics data have been deposited to the ProteomeXchange Consortium via the PRIDE partner repository with the dataset identifier PXD064077 (http://proteomecentral.proteomexchange.org/cgi/GetDataset?ID=PXD064077). Source data are provided with this paper.

Research involving human participants, their data, or biological material

Policy information about studies with human participants or human data. See also policy information about sex, gender (identity/presentation), and sexual orientation and race, ethnicity and racism.

Reporting on sex and gender	we don't have related issues
Reporting on race, ethnicity, or other socially relevant groupings	we don't have related issues
Population characteristics	we don't have related issues
Recruitment	we don't have related issues
Ethics oversight	we don't have related issues

Note that full information on the approval of the study protocol must also be provided in the manuscript.

Field-specific reporting

Please select the one below that is the best fit for your research. If you are not sure, read the appropriate sections before making your selection.

☒ Life sciences ☐ Behavioural & social sciences ☐ Ecological, evolutionary & environmental sciences

For a reference copy of the document with all sections, see nature.com/documents/nr-reporting-summary-flat.pdf

Life sciences study design

All studies must disclose on these points even when the disclosure is negative.

Sample size	• For animal work, mice were initially randomly assigned to seven groups with an initial sample size of n=24 per group. Subsets of n=6 mice per group were used for each specific endpoint assessment (endometrial repair, fertility, implantation, and receptivity marker analysis) 14 days post-treatment. The sample sizes for in vivo experiments are specified in the respective method descriptions.<br>• For in vitro experiments and proteomic analysis (LC-MS/MS), all assays were performed with at least three independent experimental repeats.
Data exclusions	No data was excluded.
Replication	All in vivo and in vitro functional and phenotypic experiments were performed in at least biological triplicate to ensure reproducibility. Similar results were obtained in at least three independent experiments.
Randomization	Materials (eg.microspheres) and tissue sections used for imaging were selected randomly, and animals were also divide into groups randomly.
Blinding	The investigators were blinded to group allocation during data collection/analysis.

Reporting for specific materials, systems and methods

We require information from authors about some types of materials, experimental systems and methods used in many studies. Here, indicate whether each material, system or method listed is relevant to your study. If you are not sure if a list item applies to your research, read the appropriate section before selecting a response.

Materials & experimental systems

n/a	Involved in the study
☐	☒ Antibodies
☐	☒ Eukaryotic cell lines
☒	☐ Palaeontology and archaeology
☐	☒ Animals and other organisms
☒	☐ Clinical data
☒	☐ Dual use research of concern
☒	☐ Plants

Methods

n/a	Involved in the study
☒	☐ ChIP-seq
☐	☒ Flow cytometry
☒	☐ MRI-based neuroimaging

Antibodies

Antibodies used	For immunostaining, PCNA (Proteintech, 60097-1-lg), Integrin β3 (Abmart, T55237), MUC1 (Abcam, ab109185), OPN (Abmart, T55333), ER (Abcam, ab32063), PR (Abcam, ab16661), HAND2 (Abcam, ab200040), Collagen Ⅰ (Cell Signaling, 66948), Collagen Ⅳ (Abcam, ab6586), α-SMA (Cell Signaling, 19245), Fibronectin (Abcam, ab32419), FoxA2 (Cell Signaling, 8186), pan CK (Abcam, ab7753), CD34 (Abcam, ab6330), ALDH1A1 (Abcam, ab52492), CD86 (Cell Signaling, 19589), Ki67 (Abcam, ab16667), and TGFβ1 (Santa Cruz, sc130348), Mannose Receptor (CD206) (Abcam, ab300621), Liver Arginase (Abcam, ab315110), iNOS (Abcam, ab283655), IL-1 β (Cohesion, CQA6579), IL-6 (Abcam, ab290735)<br>For flow cytometric, APC/Cy7-conjugated anti-mouse CD45 (BioLegend, 103115), APC-conjugated anti-mouse F4/80 (BioLegend, 123115), PE-conjugated anti-mouse CD86 (BioLegend, 159204), Brilliant Violet 421™-conjugated anti-mouse CD206 (MMR) (BioLegend, 141717).<br>For immunoblotting, GSDMD (Abmart, TA4012F), GSDMD-NT (Cohesion, CQA3563), IL-1 β (Cohesion, CQA6579), IL-18 (Cohesion, CQA2028), Caspase1-P10-P12 (Abcam, ab179515), P-smad3 (Affinity, AF3362), and GAPDH (Bioss, bs-10900R).
Validation	Validation of the commercial antibodies were done by the manufacturers.<br>Below shows the relevant information listed on the suppliers' websites for antibodies used in this article:<br>Liver Arginase (Abcam, ab315110), https://www.abcam.com/en-us/products/primary-antibodies/liver-arginase-antibody-rm1096-ab315110<br>IL-6 (Abcam, ab290735), https://www.abcam.com/en-us/products/primary-antibodies/il-6-antibody-epr23819-103-ab290735<br>PCNA (Proteintech, 60097-1-lg), https://www.ptglab.com/products/PCNA-Antibody-60097-1-Ig.htm<br>Integrin β3 (Abmart, T55237), http://www.ab-mart.com.cn/products_1_P_T55237.html<br>MUC1 (Abcam, ab109185), https://www.abcam.com/products/primary-antibodies/muc1-antibody-ab109185.html<br>OPN (Abmart, T55333), http://www.ab-mart.com.cn/products_1_P_T55333.html<br>ER (Abcam, ab32063), https://www.abcam.com/products/primary-antibodies/er-alpha-antibody-ab32063.html<br>PR (Abcam, ab16661), https://www.abcam.com/products/primary-antibodies/pr-antibody-ab16661.html<br>HAND2 (Abcam, ab200040), https://www.abcam.com/products/primary-antibodies/hand2-antibody-ab200040.html<br>Collagen 1 (CST, 66948), https://www.cellsignal.cn/products/primary-antibodies/collagen-type-i-e9i5k-mouse-mab/66948<br>Collagen 4 (Abcam, ab6586), https://www.abcam.com/products/primary-antibodies/collagen-iv-antibody-ab6586.html<br>α-SMA (CST, 19245), https://www.cellsignal.cn/products/primary-antibodies/alpha-smooth-muscle-actin-d4k9n-xp-rabbit-mab/19245<br>Fibronectin (Abcam, ab32419), https://www.abcam.com/products/primary-antibodies/fibronectin-antibody-ab32419.html<br>FoxA2 (CST, 8186), https://www.cellsignal.cn/products/primary-antibodies/foxa2-hnf3beta-d56d6-xp-rabbit-mab/8186<br>pan CK (Abcam, ab7753), https://www.abcam.com/products/primary-antibodies/pan-cytokeratin-antibody-ae1ae3-ab7753.html<br>CD34 (Abcam, ab6330), https://www.abcam.com/products/primary-antibodies/cd34-antibody-qbend10-ab6330.html<br>ALDH1A1 (Abcam, ab52492), https://www.abcam.com/products/primary-antibodies/aldh1a1-antibody-ab52492.html<br>CD86 (CST, 19589), https://www.cellsignal.cn/products/primary-antibodies/cd86-d6y5h-rabbit-mab/19589<br>Mannose Receptor (CD206) (Abcam, ab300621), https://www.abcam.com/en-us/products/primary-antibodies/mannose-receptor-antibody-epr25215-277-ab300621<br>Ki67 (Abcam, ab16667), https://www.abcam.cn/products/primary-antibodies/ki67-antibody-sp6-ab16667.html<br>TGFβ1 (Santa Cruz, sc130348), https://www.scbt.com/zh/p/tgf-beta-1-antibody-sc-130348<br>GSDMD (Abmart, TA4012F), http://www.ab-mart.com.cn/products_1_P_TA4012F.html<br>P-smad3 (Affinity, AF3362), https://www.affbiotech.com/item/af3362.html<br>IL-1β (Cohesion, CQA6579), http://www.cohesionbio.com/show-168-696.html<br>GSDMD-NT (Cohesion, CQA3563), http://www.cohesionbio.com/show-168-529.html<br>IL-18 (Cohesion, CQA2028), http://www.cohesionbio.com/show-168-295.html<br>Caspase1-P10-P12 (Abcam, ab179515), https://www.abcam.com/products/primary-antibodies/caspase-1-p10-p12-antibody-ab179515.html<br>GAPDH (Bioss, bs-10900R), https://www.bioss.com/anti-gapdh-rabbit-polyclonal-antibody-bs-10900r.html<br>APC/Cy7-conjugated anti-mouse CD45 (BioLegend, 103115), https://www.biolegend.com/de-at/products/apc-anti-mouse-cd45-

antibody-97?GroupID=BLG6837
APC-conjugated anti-mouse F4/80 (BioLegend, 123115), https://www.biolegend.com/de-at/products/apc-anti-mouse-f4-80-antibody-4071
PE-conjugated anti-mouse CD86 (BioLegend, 159204), https://www.biolegend.com/de-at/products/pe-anti-mouse-cd86-antibody-18945
Brilliant Violet 421™-conjugated anti-mouse CD206 (MMR) (BioLegend, 141717), https://www.biolegend.com/de-at/products/brilliant-violet-421-anti-mouse-cd206-mmr-antibody-8638

Eukaryotic cell lines

Policy information about cell lines and Sex and Gender in Research

Cell line source(s)	Human endometrial stromal cells (hESC, CRL-4003) were purchased from ATCC. The human endometrial adenocarcinoma cell line (Ishikawa) was provided by Prof. Zengming Yang (Guizhou University). Human umbilical vein endothelial cells (hUVEC) were provided by Dr. Jianbing Liu (Shanxi Medical University).
Authentication	The hESC line was authenticated by ATCC via STR profiling. The Ishikawa and hUVEC lines were verified in-house by assessing cellular morphology and expression of specific markers.
Mycoplasma contamination	All cell lines tested negative for mycoplasma contamination.
Commonly misidentified lines (See ICLAC register)	No commonly misidentified cell lines were used.

Animals and other research organisms

Policy information about studies involving animals; ARRIVE guidelines recommended for reporting animal research, and Sex and Gender in Research

Laboratory animals	2-month-old ICR female mice
Wild animals	The study did not involve wild animals.
Reporting on sex	only females were used in this study
Field-collected samples	The study did not involve samples collected from the field.
Ethics oversight	All experimental protocols involving animals received approval from the Animal Ethics Committee of Shanxi Medical University (Approval No: SYDL2023018) and adhered strictly to relevant ethical guidelines.

Note that full information on the approval of the study protocol must also be provided in the manuscript.

Plants

Seed stocks	we don't have related issues
Novel plant genotypes	we don't have related issues
Authentication	we don't have related issues

Flow Cytometry

Plots

Confirm that:

- [x] The axis labels state the marker and fluorochrome used (e.g. CD4-FITC).
- [x] The axis scales are clearly visible. Include numbers along axes only for bottom left plot of group (a 'group' is an analysis of identical markers).
- [x] All plots are contour plots with outliers or pseudocolor plots.
- [x] A numerical value for number of cells or percentage (with statistics) is provided.

Methodology

Sample preparation	Uterine tissues (n = 3 biologically independent animals per group) were harvested, minced into small fragments (~1–2 mm^3),

and dissociated using a sequential enzymatic digestion protocol. Tissues were incubated in HBSS containing 1% trypsin, 6 mg/mL dispase, and 1 mg/mL DNase I for 1 h at 4 °C, 1 h at room temperature, and 10 min at 37 °C, followed by secondary digestion with 0.15 mg/mL collagenase I for 35 min at 37 °C with gentle agitation. The reaction was neutralized with FACS buffer (DPBS with 2% FBS and 1 mM EDTA), and suspensions were filtered through a 70-µm strainer. Single-cell suspensions (1–2 × 10^6 cells) were stained with the Zombie Aqua™ Fixable Viability Kit to exclude dead cells and incubated with anti-mouse CD16/32 to block Fc receptors. Surface staining was performed on ice for 30 min using fluorochrome-conjugated antibodies against CD45, F4/80, and CD86. Cells were subsequently fixed with 4% paraformaldehyde, permeabilized with 0.2% Triton X-100, and stained intracellularly with anti-CD206 for 35 min. Data were acquired on a BD FACSAria™ Fusion Flow Cytometer and analyzed using FlowJo software (v10.8).

Instrument

BD FACSAria™ Fusion Flow Cytometer

Software

FlowJo software (v10.8)

Cell population abundance

No cell sorting was performed for downstream analysis. The abundance of specific cell populations (M1-like and M2-like macrophages) was quantified via flow cytometry analysis and is reported as the percentage of the parent CD45+F4/80+ population in the relevant figures and results sections.

Gating strategy

The gating strategy was performed as follows: (1) exclusion of debris based on forward scatter (FSC) and side scatter (SSC); (2) exclusion of doublets using FSC-H versus FSC-A; (3) identification of live cells (Zombie Aqua-negative); (4) selection of leukocytes ($CD45^+$); and (5) identification of macrophages ($F4/80^+$). Within the $F4/80^+$ population, M1-like ($CD86^+$) and M2-like ($CD206^+$) phenotypes were quantified based on fluorescence minus one (FMO) controls. Representative gating strategies are provided in Supplementary Fig.8. Detailed antibody information is listed in Supplementary Table 1.

☒ Tick this box to confirm that a figure exemplifying the gating strategy is provided in the Supplementary Information.
